# Novel pyroptosis-independent functions of gasdermins

**DOI:** 10.1038/s41392-022-00991-3

**Published:** 2022-04-22

**Authors:** Ziran Qin, Fangfang Zhou, Long Zhang

**Affiliations:** 1 Zhejiang University City College, Hangzhou, China; 2 Institutes of Biology and Medical Science, Soochow University, 215123 Suzhou, China; 3 MOE Laboratory of Biosystems Homeostasis and Protection and Innovation Center for Cell Signaling Network, Life Sciences Institute, Zhejiang University, 310058 Hangzhou, China

**Keywords:** Cell biology, Biochemistry

Recently, Rana et al. and Zhang et al. published two studies illustrating the important regulatory roles of gasdermin B (GSDMB) and -D (GSDMD) in inflammatory bowel disease (IBD) and intestinal immune homeostasis maintenance, respectively.^1,2^ Their findings collectively indicate that the gasdermins (GSDMs) can play a crucial role in restoring epithelial barrier function and shaping gut mucosal homeostasis. Moreover, neither of the functions of GSDMs revealed by these two studies are related to pyroptosis, which may provide new insights into the non-pyroptosis-dependent functions of GSDM proteins.

The GSDM family has evolved into six gene clusters (*GSDMA-E* and pejvakin [*PJVK*]), primarily described for their role in pyroptosis, and GSDM proteins are characterized by a unique N-terminal domain (N-GSDM). Except for PJVK, the N-GSDM domain can permeabilize the plasma membrane.^3^

Goblet cells in the intestinal epithelium form the mucus layer by secreting mucus proteins, which limit gut microbial colonization and invasion and play a pivotal role in maintaining gut homeostasis. If the mucus layer disappears, direct contact of bacteria with the epithelium results in IBD, which is characterized by chronic, relapsing inflammation.^1^ To date, there is no known cure for IBD; instead, current treatment modalities aim to control the symptoms and achieve long-term remission. Furthermore, the regulatory mechanisms underlying mucus layer formation remain largely unknown.

A genome-wide association study showed that GSDMB is associated with human IBD.^4^ To determine the role of GSDMB in IBD, Rana et al. investigated the expression pattern of GSDMB using different databases from patients with IBD. They found increased expression of GSDMB (mainly located in epithelial cells) in inflamed lesions of patients with IBD compared to healthy controls. Interestingly, subsequent ribonucleic acid sequencing and Gene Ontology analyses showed that the upregulated gene signaling pathways in cells with increased GSDMB expression were mainly associated with cell proliferation, migration, and adhesion, rather than pyroptosis. To validate these unexpected results, the authors treated different intestinal epithelial cell lines with a GSDMB activator and found that wild-type intestinal epithelial cell lines were more potent in proliferation, migration, and cell adhesion than mutant or GSDMB-deficient cell lines. Subsequently, they investigated a battery of adhesion-related molecules to explore the potential mechanisms found above and observed that in mutant or GSDMB-deficient cell lines, the phosphorylation specificity of FAK associated with cell adhesion decreased with a decrease in PDGFA, a polypeptide growth factor demonstrated to phosphorylate FAK. Therefore, GSDMB is increased in IBD and regulates epithelial structure/repair independently of pyroptosis^1^. Almost at the same time, Zhang et al. reported that GSDMD has an important regulatory role in maintaining intestinal immune homeostasis. To explore the significance of the expression and activation of GSDMD in intestinal epithelial cells, Zhang et al. conditionally deleted GSDMD in intestinal epithelial cells by generating Villin-cre^+^ GSDMD^f/f^ (GSDMD^ΔIEC^) mice; GSDMD^ΔIEC^ mice exhibited more severe bacterial infection. Combining histological staining and electron microscopy with *Citrobacter rodentium* quantification experiments, they were surprised to find that the colonic mucus layer of GSDMD^ΔIEC^ mice had almost disappeared, which resulted in a consequent massive adherence of gut commensals directly to the gut epithelial surface. Mechanistically, quantitative proteomic data and GO analysis of colonic epithelial cells indicated that the secretory vesicle efflux pathway was significantly inhibited in GSDMD^ΔIEC^ mice. Among these, scinderin, a cytoskeletal regulatory protein, was most significantly downregulated. Using a GSDMD-deficient human colonic goblet cell line, investigators showed that GSDMD pores are required for Ca^2+^ influx, which can drive scinderin function. Scinderin, in turn, drives F-actin disassembly, which ultimately promotes the efflux of mucus vesicles and the formation of the intestinal mucus layer^2^ (Fig. 1).

**Fig. 1:**
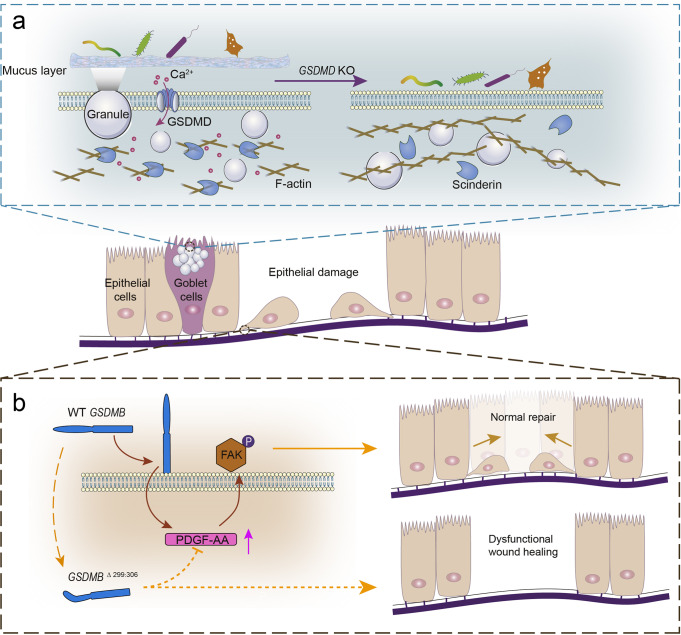
Function of gasdermins in intestinal epithelial restitution and immune homeostasis. **a** In goblet cells, GSDMD pores mediate calcium ion influx, thereby activating scinderin-mediated F-actin disassembly and mucus secretion to form the mucus layer. GSDMD deficiency leads to the absence of the mucus layer and direct bacterial contact with the epithelium. **b** In epithelial cells, full-length GSDMB elevates intracellular PDGF-AA and thus promotes FAK phosphorylation, resulting in enhanced cell proliferation and migration. The carriage of GSDMB mutations leads to a defect in the repair capacity of epithelial cells

Although current literature suggests an undeniable link between GSDMB and genetic susceptibility to IBD, functional studies are limited by the lack of in vivo models. Future studies utilizing animal models that transgenically express human GSDMB may clarify its role in an in vivo setting.^1^ Notably, Rana et al. demonstrated that GSDMB regulates cell migration and adhesion functions by affecting the expression of PDGFA, but the specific molecular mechanism by which GSDMB regulates PDGFA remains unknown. In addition, GSDMs generally function through the activation of the N-GSDM domain and the pore-forming mechanism, so what biological function does the translocation of full-length GSDMB to the cytoplasmic membrane play? These are important potential research directions for the future.

Zhang et al. also showed that GSDMD uses a common membrane-targeting mechanism to form pores which lead to lytic cell death, membrane repair, or granule exocytosis in different physiological contexts.^2^ This raises a crucial and intriguing question of how different cell types respond to these structurally similar GSDMD pores in different ways. Zhang et al. hypothesized that differences in the number and size of GSDMD pores between different cell types and populations may be the cause. Future work in structural biology may help address this question. Furthermore, we conjecture that there are other different proteins that assist in the different functions of GSDMD pores. Extensive and systematic work needs to be done by researchers in the future to explain these doubts.

Taken together with the study by Rana et al., these two studies demonstrate novel functions of GSDMs in pyroptosis-independent processes, particularly, in the maintenance of immune homeostasis and epithelial reconstitution in the intestine. In addition, Rana et al. identified methotrexate as a non-inflammatory stimulus capable of upregulating GSDMB, and Zhang et al. successfully established an in vitro colon organoid culture system by three-dimensional printing.^1,2^ These findings provide strong support for the further development of drugs targeting GSDMs and optimization of therapeutic strategies for the treatment of IBD. It is worth noting that more and more studies have been conducted on the pyroptosis-independent functions of GSDMs. For example, GSDMA is frequently silenced in gastric cancer and has tumor growth factor-β-regulated growth-inhibition activity. Genetic mutations of GSDME and DFNB59 (pejvakin) cause nonsyndromic hearing loss in humans.^5^ These findings suggest that we are only at the tip of the iceberg in understanding and appreciating the biological and pathological functions of GSDMs.^5^

## References

[1] Rana N (2022). GSDMB is increased in IBD and regulates epithelial restitution/repair independent of pyroptosis. Cell.

[2] Zhang J (2022). Epithelial gasdermin D shapes the host-microbial interface by driving mucus layer formation. Sci. Immunol..

[3] De Schutter E (2021). Punching holes in cellular membranes: biology and evolution of gasdermins. Trends Cell Biol..

[4] Jostins L (2012). Host-microbe interactions have shaped the genetic architecture of inflammatory bowel disease. Nature.

[5] Shi J, Gao W, Shao F (2017). Pyroptosis: gasdermin-mediated programmed necrotic cell death. Trends Biochem Sci..

